# Supplementary material for: A Wnt5a-Cdc42 axis controls aging and rejuvenation of hair-follicle stem cells
Source: Aging (Albany NY). 2021 Feb 25;13(4):4778–93. doi: 10.18632/aging.202694 (PMC7950224; doi:10.18632/aging.202694)
Supplement: Supplementary Figures [file aging-13-202694-s001.pdf]

SUPPLEMENTARY FIGURES

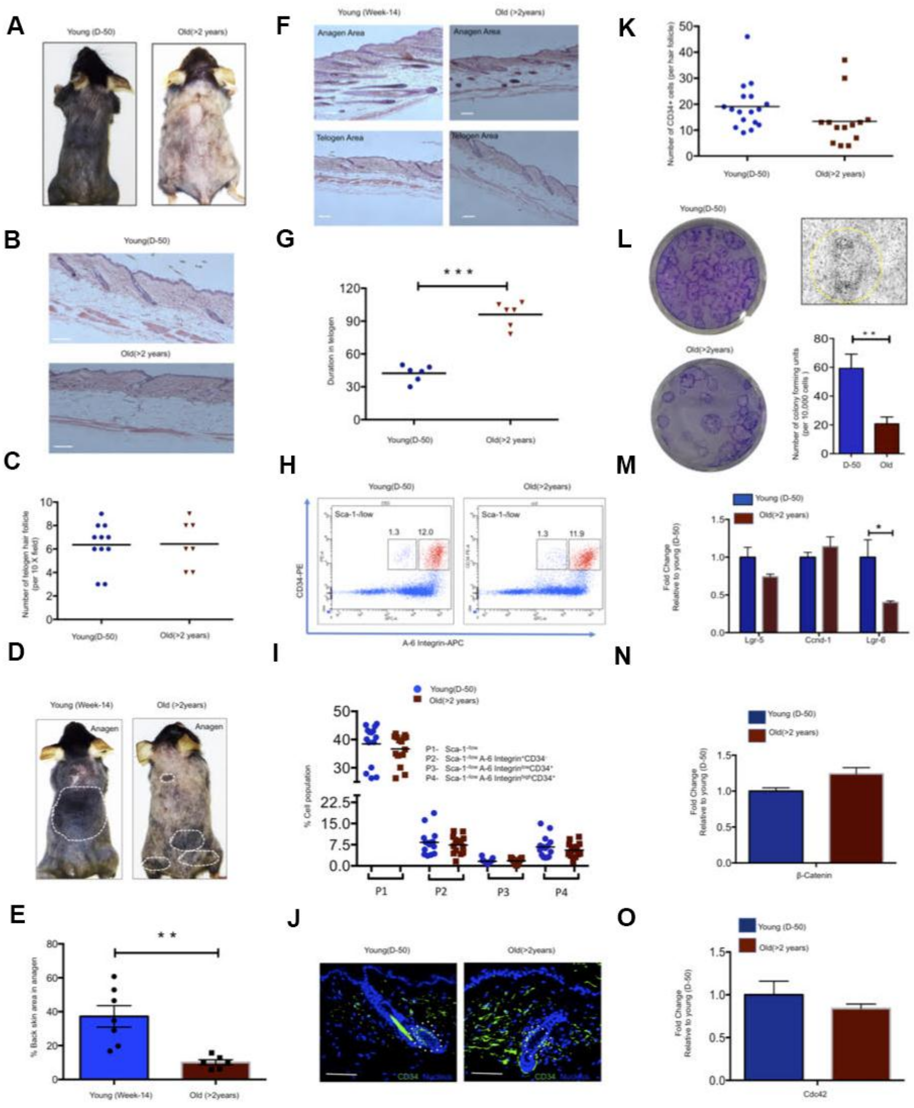

Supplementary Figure 1. Functional decline of aged HFSC. (**A**) Young (D-50) and old (>2years) mice in telogen stage, hair shaved, (**B**) H&E staining of a longitudinal section of back skin from young (D-50) and old (>2years) mice (miniaturized hair follicle), scale bar = 100µm (**C**) Number of telogen hair follicle counted per 10X field picture of telogen mice skin of young D-50 and old >2 years, N≥7 (**D**) Morphology of young (week-14) and old(>2years) mice in anagen stage after hair shaving, anagen area are encircled by white lines (**E**) measurement of anagen areas from young (week-14) and old(>2years) mice and represented as percentage of complete back skin area, N≥6 (**F**) Representative picture of H&E stained LS of back skin from, anagen area(upper panel), telogen area (lower panel) of young (week-14) and old(>2years) mice, yellow yarrow shows hyper proliferated bulge morphology, Scale bar = 100µm (**G**) Duration of telogen in young(D-50) and old mice(>2years) showing increase in duration of old HFSC in telogen stage, N=6 (**H**) FACS analysis of Sca-1-/low fraction to detect different cell population in young and old mice (**I**) Percentage of different cell population in epidermal cell suspension of skin from D-50 and Old mice showing no change in HFSC number, N≥14 (**J**) Immunofluorescence images with HFSC marker CD34 (green and nucleus (blue) in LS of telogen skin from young(D-50) and old mice N≥3, Scale bar = 50µm (**K**) Quantification of number of CD34+ cells counted per telogen hair follicle in young(D-50) and old (>2years) telogen hair follicle, (**L**) Representative picture of colony forming units from FACS sorted HFSC from young and old mice in telogen(left panel),Representative morphology of colonies obtained during CFU analysis (right upper panel), Quantification of CFU per 10,000 HFSC plated from young and old mice indicating decrease in colony forming units in old HFSC (right lower panel), (**M**) Transcript levels of canonical Wnt target genes (Lgr-5, Ccnd-1 and Lgr-6) (**N**) ß-catenin (**O**) Cdc42 in young and old HFSC N≥3,*P<.05, **P<.01, ***P<.001 (paired student's t test). Error bars represent s.e.m.

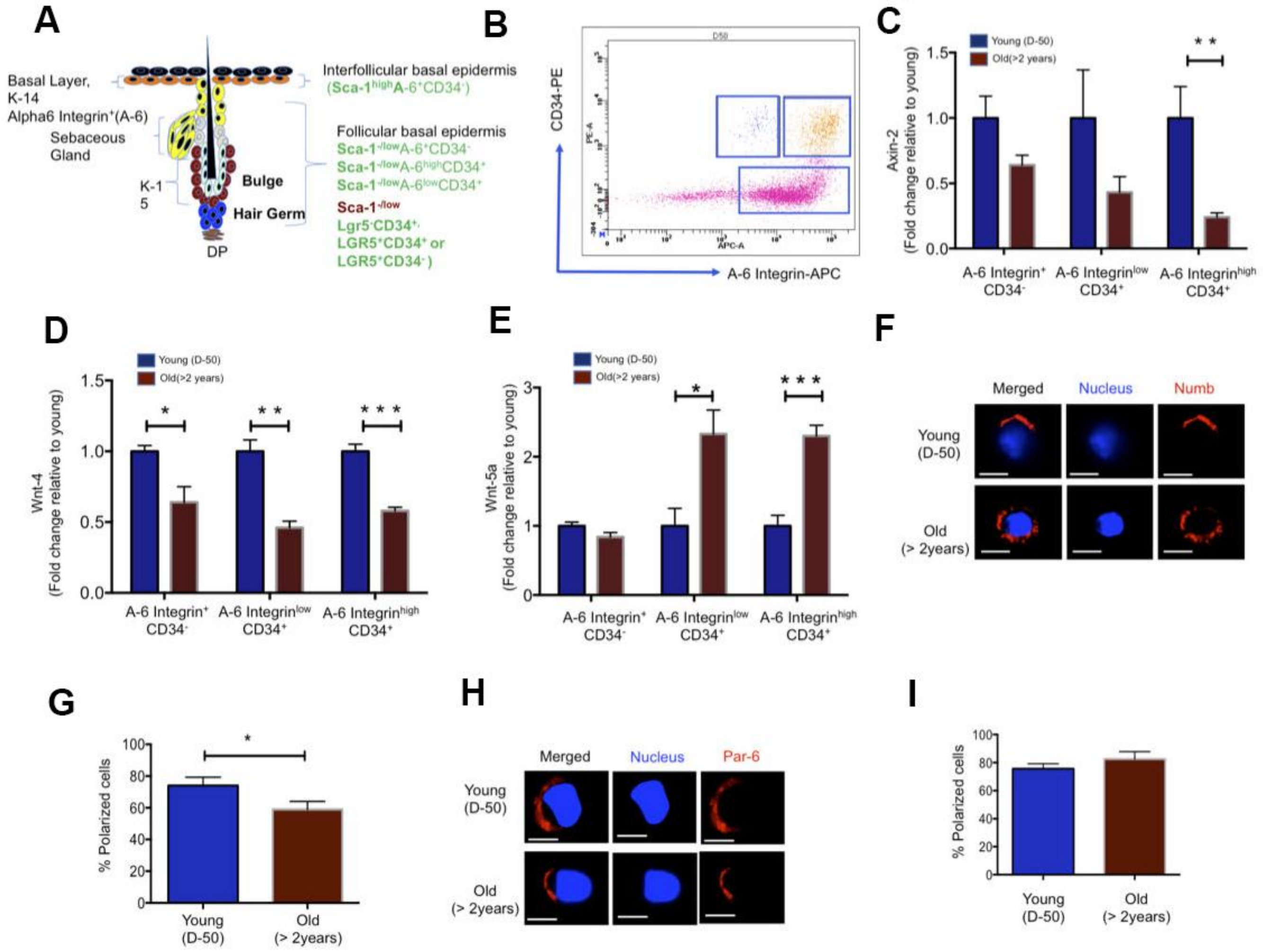

Supplementary Figure 2. Increase in Wnt5a expression upon aging is specific to a CD34$^+$ cell population. (**A**) Graphical representation of the structure of the hair follicle and location of distinct types of stem and progenitor cells (**B**) dot plot of FACS analyses on Sca-1$^{-/low}$ keratinocytes showing three different cell (A-6$^+$CD34$^-$, A-6^{low}CD34$^+$ and A-6 ^{high} CD34$^+$) populations obtained during FACS sorting (**C**) transcript levels of Axin-2 (**D**) Wnt4 and (**E**) Wnt5a in young and old A-6$^+$CD34$^-$,A-6^{low}CD34$^+$ and A-6 ^{high} CD34$^+$ cells, N≥3 (**F**) Representative picture of the distribution of Numb (red) in young and old HFSC, immunofluorescence, Scale bar =5μm (**G**) Percentage of cells polar for Numb in young and old HFSC, N≥3 (**H**) Distribution of Par-6 (red) in young and old HFSC, immunofluorescence, Scale bar =5μm (**I**) Percentage of cells polar for Par-6 in young and old HFSC, N≥4, *P<.05, **P<.01, ***P<.001. (paired student's t test). Error bars represent s.e.m.

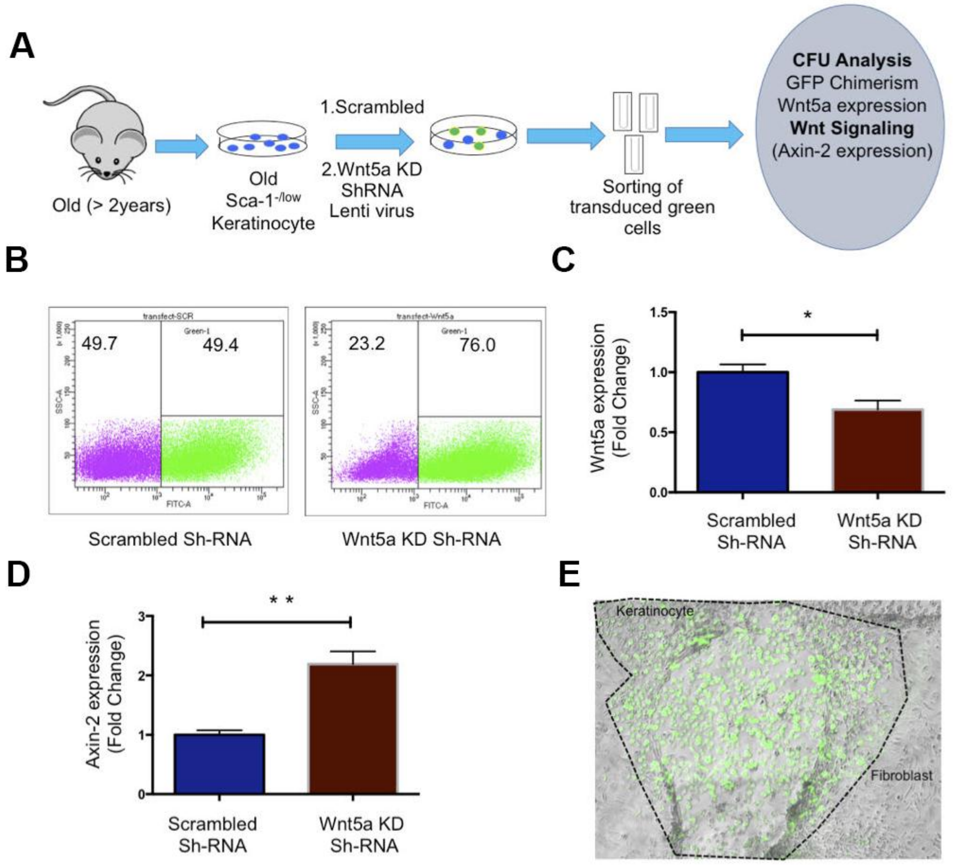

Supplementary Figure 3. Suppression of expression of Wnt5a in old HFSC induces a young-like phenotype. (**A**) Experimental setup (**B**) Representative FACS plot showing percentage of transduced cell in scrambled shRNA and Wnt5a knock-down shRNA transduced cells on day 7 post transduction, N≥3 (**C**) level of expression of Wnt5a in scrambled shRNA and Wnt5a knock-down shRNA transduced cells on day 7 post transduction, N≥3 (**D**) Expression of canonical Wnt-target genes in scrambled shRNA and Wnt5a knock-down shRNA transduced cells on day 7 post transduction, N≥3 (**E**) Representative picture of green colony formed by a transduced old Sca-1-/low keratinocyte, surrounding fibroblast feeder cells are non-green, N≥3. *P<.05, **P<.01 (paired student's t test). Error bars represent s.e.m.

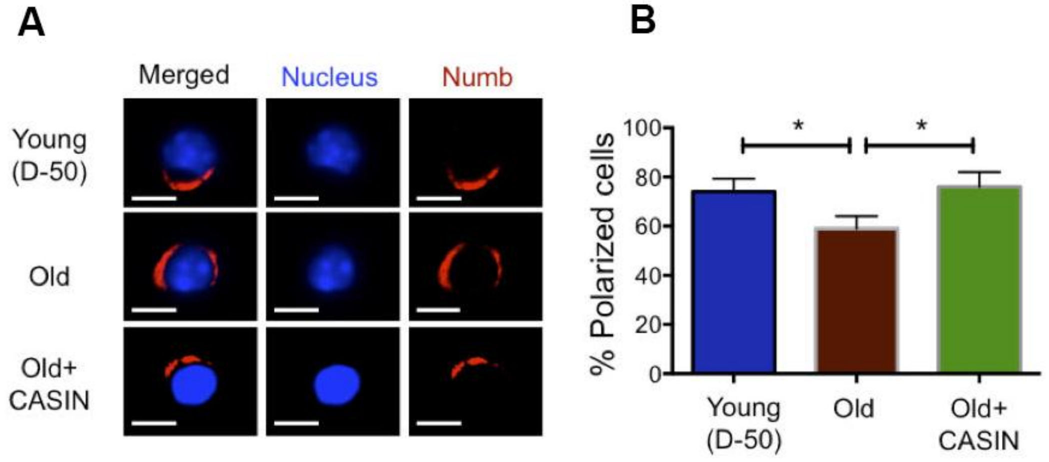

Supplementary Figure 4. CASIN treatment of aged HFSCs restores a youthful level of cell polar for Numb. (**A**) Representative picture of Numb (red) in young, old and CASIN treated HFSC, Immunofluorescence, N≥3 (**B**) Percentage of cells polar for Numb in young, old and old CASIN treated HFSCs, N≥3. Scale bar =5µm, *P<.05. (paired student's t test). Error bars represent s.e.m.
